# Recruitment and retention of young adults with sickle cell disease

**DOI:** 10.1016/j.hctj.2026.100133

**Published:** 2026-03-14

**Authors:** Tanisha Belton, Lane Carbaugh, Evelyn Stevens, Abigail Seide, Bianca Ferreira, Olivia Teng, Laura Bennett, Amanda Pfeiffer, Mark Ferreira, Amy Shova, Robin Arens, Desireé N. Williford, Banu Aygun, Abena Appiah-Kubi, Nataly Apollonsky, Donna Boruchov, Omar Niss, Kim Smith-Whitley, Sophia Jan, Caren M. Steinway

**Affiliations:** a Children’s Hospital of Philadelphia, United States; b Northwell Health, United States; c Donald and Barbara Zucker School of Medicine at Hofstra/Northwell, United States; d Drexel University College of Medicine, United States; e Cincinnati Children’s Hospital Medical Center, United States; f Connecticut Children’s, United States; g University of Cincinnati College of Medicine, United States; h St. Christopher’s Hospital for Children, United States; i University of Connecticut Health, United States; j University of Pennsylvania, United States

**Keywords:** Recruitment, Retention, Sickle cell disease, Adolescents and young adults

## Abstract

**Background:**

Research has illuminated the challenges of recruiting and retaining adolescents and young adults (AYA) in research studies due to environmental, community, and personal factors. The Community Health Workers and Mobile Health for Emerging Adults Transitioning Sickle Cell Disease Care (COMETS) Study is a RCT comparing the effectiveness of two self-management support interventions, Community Health Workers (CHW) and Mobile Health (mHealth), versus enhanced usual care to improve health-related quality of life for transitioning AYA with sickle cell disease (SCD). The aim of this paper is to describe recruitment and retention strategies used in the COMETS study.

**Methods:**

Recruitment and retention strategies that were identified prioritized the complex relationships between AYA with SCD and their environments. AYA (17 and older) were approached for study participation across five SCD centers in NY, PA, OH, and CT. Study team members approached patients to complete surveys at 6-, 12-, and 18- months after study enrollment.

**Results:**

Of the 496 young adults approached, 405 were enrolled and 375 were randomized (median age=18 years, range=17–28). Enrolled subjects evenly identified as male (49%) and female (50%). Most participants identified as Black or African American (95%) and 8.2% of participants identified as Hispanic/Latino. Retention rates were: 82% at 6 months, 82% at 12 months and 77% at 18 months.

**Conclusions:**

Implementing recruitment and retention strategies that take into consideration participant environmental, community, and personal factors are imperative for success. Study teams should be prepared for ongoing refinement of strategies based on participant and collaborator feedback.

## 1 Introduction

When developing the design and implementation of a randomized control trial (RCT), the successful and timely recruitment of the required patient population is the pinnacle of the priority list. Adolescents and young (AYA) adults with sickle cell disease (SCD) face significant challenges during the transition from pediatric care to adult focused care.^1^ Interventions that address self-management and support AYA with SCD during this vulnerable period are critical, yet recruitment and retention of AYA remains a challenge for interventions regardless of therapeutic area.^2^, ^3^, ^4^

It has been reported that in clinical trials involving patients with SCD, 57% of terminated trials are due to a lack of enrollment.^5^ Low enrollment and attrition have been attributed to limited recruitment planning, inconsistent communication, and missed opportunities for engagement during routine clinical encounters.^6^ Effective recruitment is necessary to demonstrate efficacy of a given intervention and reduce cost implications associated with delayed recruitment.^7^ Once the hurdle of recruitment is addressed, researchers face a secondary challenge to retain participants to ensure valid data and sufficient power for data analysis. Challenges are compounded during times of uncertainty, such as a global pandemic.

The Community Health Workers and Mobile Health for Emerging Adults Transitioning Sickle Cell Disease Care (COMETS) Study is a multi-center randomized controlled trial (RCT) comparing the effectiveness of two self-management support interventions, Community Health Workers (CHW) and Mobile Health (mHealth), versus Enhanced Usual Care (EUC) to improve health-related quality of life for transitioning AYA with SCD.^8^ Conducted across five pediatric SCD centers, the study incorporated multiple recruitment and retention strategies designed to address the individual, interpersonal, organizational, and community level-influences on research participation.

The social ecological model (SEM)^9^ helps understand the intersectionality of individual level behaviors with the influence of social and environmental factors. SEM is widely used to understand health behaviors specifically recognizing that AYA live within systems where personal and environmental factors may influence health behaviors, such as participation in clinical trials. Many studies employ the use of the social ecological model to help to anticipate and contextualize the needs of the given patient population. By examining these factors on a deeper level, researchers can anticipate the need of multiple strategies to recruit potential participants. Thus, the social ecological model can be a useful framework for developing effective recruitment and retention strategies for AYA with SCD. Effective recruitment is necessary to demonstrate efficacy of a given intervention and reduce cost implications associated with delayed recruitment.^7^

The aim of this paper is to describe the recruitment and retention strategies implemented in the COMETS study and associated recruitment and retention outcomes. By detailing the workflows, stakeholder engagement, communication approached, and adaptations employed across study sites, this manuscript seeks to inform future trials engaging AYA with SCD and other populations navigating critical healthcare transitions.

## 2 Methods

### 2.1 Study design and setting

The COMETS study is a multicenter randomized controlled trial conducted from January 2019 through June 2024 across five SCD centers in New York, Pennsylvania, Ohio and Connecticut. AYA aged 17 years and older were approached for study participation. Following enrollment and randomization, participants were asked to complete surveys at 6-, 12-, and 18-month follow-up intervals. Several strategies were implemented throughout the planning and conduct of the COMETS study to ensure recruitment and retention remained a high priority.

### 2.2 Stakeholder advisory committee (SAC)

**Fig. 1:**
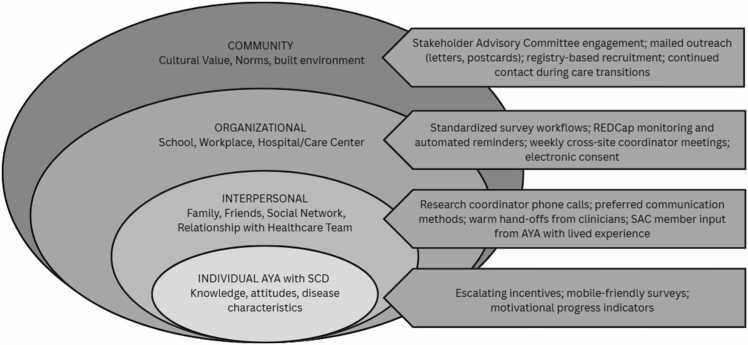
Alignment of recruitment and retention strategies to the social ecological model (SEM).

A stakeholder advisory committee (SAC) was established at study inception to inform recruitment, retention, and intervention design, which were guided by the SEM (See Fig. 1).^9^ SAC members were purposefully recruited from a range of community-based organizations, advocacy groups, policy makers, and clinical settings with demonstrated experience supporting AYA with SCD in the communities where the study was implemented. Importantly, the committee included AYA with lived experience with SCD.

The SAC convened quarterly throughout the study period. Members provided feedback on recruitment messaging, compensation amounts (in consideration of the survey lengths [45–90 min long] and mobile health app usage), and the design of both interventions (community health worker and mHealth app) to ensure they were user-friendly and appealing to participants. The SAC continued to advise the study team during the COVID-19 pandemic, contributing to adaptations in recruitment and retention strategies.

### 2.3 Recruitment strategies

Recruitment occurred between January 2019 and December 2022, with a recruitment pause from May 2019 until July 2020 due to delays in mobile health application development and the COVID-19 pandemic. Recruitment strategies were designed to address individual, organizational, and community-level influences on research participation among AYA with SCD. Fig. 1 shows the alignment of strategies to the SEM. Participants were identified through clinic schedules and SCD registries and approached using multiple modalities, including in-person recruitment during outpatient visits and inpatient admissions, as well as telephone outreach. At study inception, mailed and emailed recruitment materials, including a study letter, flyer, and a recruitment video, were distributed. Weekly meetings between

Research Coordinators (RC) across the five participating sites and study program managers were held to monitor recruitment progress, share challenges, and refine strategies in real time.

RC’s reviewed study materials with participants to ensure informed participation and minimize burden through a low-risk protocol that did not involve medications or procedures, eliminated required in-person study visits, and provided compensation for participation.

In response to the constraints imposed by the COVID-19 pandemic, telephone recruitment and electronic consent (e-consent) were incorporated as additional strategies. While e-consent is not a novel approach, the pandemic catalyzed the use of e-consenting which has contributed to improving patient understanding, reduced enrollment time, and improved data accuracy.^10^

### 2.4 Survey completion workflow

Given the length and longitudinal nature of study surveys, a structured workflow was developed to support timely survey completion. Participants were provided two weeks to complete the baseline surveys to maintain enrollment. Recognizing that AYA are balancing school, work, family, friends, and other obligations, participants were afforded the ability to pause and resume surveys within each survey window.

At baseline and after consent, participants completed a brief demographic survey. This included a brief, five-minute form, either in person or by phone, as the final step of enrollment. This process (1) captured each participant’s preferred method of contact and (2) introduced them to the survey platform with a RC available to address questions. RCs monitored survey progress in REDCap^11^, ^12^ and set automated reminders every two days, with a maximum of five reminders using the participant’s preferred method of contact. Five to seven days post-enrollment, RCs called participants to notify them of the pending survey expiration and to reset reminders as needed. If contact was made, reminders were often given during the call.

For the 6-month and 12-month follow-up surveys, initial reminders were sent every five days, aligning with each participant's' preferred method of contact for the maximum of five reminders, with frequency increasing as the deadline approached. Participants had up to six months to complete each follow-up survey. For the 18-month survey, participants were given two months to complete the survey, with reminders sent every three days initially and increasing to every two days at the midpoint, supplemented by personalized phone calls. After the first reminder cycle, RC’s alternated reminder methods (e.g. from email to text or vice versa).

Additional procedures were implemented to support survey completion across varying participant schedules and clinical contexts. RCs set personal calendar reminders to monitor survey progress approximately three times per week and adjusted reminder timing based on participant availability, including sending reminders after 5:00PM, on weekends, and during school holidays. When participants had upcoming clinic appointments, RCs coordinated with clinical providers to confirm contact information, and, when feasible, met participants in person to provide reminders and survey assistance. Laptops and tablets were made available during clinic visits to facilitate survey completion in waiting areas or after appointments.

To avoid overlap across survey intervals, each follow-up survey was linked to a defined completion window, and subsequent surveys were not released until the prior survey window had closed.

### 2.5 Communication, accessibility, and incentives

While retention strategies are a crucial element for any RCT, retention strategies are not often reported in published research.^7^ The COMETS study implemented a multifaceted approach, including flexible survey completion options, automated and personalized reminders, and incentives that increased as participants advanced through the study timeline. At enrollment, RCs collected comprehensive contact information, including secondary contact when available. Communication strategies included phone calls, emails, text messages, mailed holiday cards, and reminder postcards, which also served to confirm updated contact information over time. This immediate engagement encouraged continuity, as participants typically completed additional survey components independently afterward. To maintain engagement, surveys included motivational markers, such as relevant memes and milestone reminders, indicating progress and reinforcing the incentive structure.

Participants received compensation for completing surveys at baseline (US $50), 6 months (US $75), 12 months (US $100), and 18 months (US $150). Participants randomized to the mobile health arm could earn up to an additional $11 for engaging with the iManage SCD application over the first six months with the app. Payments are managed through ClinCards, a secure, reloadable debit card.2.6 Ethical Considerations

The COMETS study was funded in September 2017, IRB approval was received in June 2018 (IRB #18–015106) at the prime institution, The Children’s Hospital of Philadelphia and submitted to each participating site for site-level approach. This study was registered in August 2018 (NCT03648710). Written informed consent was obtained from all participants prior to enrollment, with electronic consent utilized when in-person consent was not feasible.

To facilitate interpretation of the recruitment and retention strategies summarized in Table 1, strategies were retrospectively categorized as either high yield or supportive. Strategies were classified as high yield when their implementation could be directly linked to measurable recruitment or retention outcomes, such as the number of participants enrolled following specific outreach approaches or coordinator contact. Strategies were classified as supportive when they were designed to facilitate participant engagement or reduce barriers to study participation but were not directly associated with a quantifiable recruitment or retention metric. These classifications were informed by study-level recruitment and retention data, including patterns observed in site-specific retention rates and recruitment workflows across participating centers.

**Table 1:** Demographic and clinical characteristics.

**Factor**	**Statistics** **(N = 375)**
**Age**	18 [17,28]
**Race**	
Black or African American	356 (95)
White	9 (2.3)
Asian	2 (0.50)
American Indian or Alaskan Indian	4 (1.0)
Native Hawaiian/Other Pacific Islander	2 (0.5)
Other	19 (5.1)
Prefer Not to Say	3 (0.80)
**Hispanic/Latino**	
Yes	31 (8.2)
No	340 (91)
Prefer Not to Say	4 (1.1)
**Assigned Sex at Birth**	
Male	187 (50)
Female	187 (50)
Prefer Not to Say	1 (0.30)
**Gender Identity**	
Male	184 (49)
Female	187 (50)
Other	2 (0.40)
Prefer Not to Say	2 (0.40)
**SCD Severity**	
Moderate	161 (43)
Severe	214 (57)
**Employment**	
Part-time Student	38 (10)
Full-time Student	173 (46)
Working Part-time	101 (27)
Working full-time	41 (11)
Unemployed	116 (31)
Other	8 (2.1)
Prefer Not to Say	6 (1.5)
**Currently Living With**	
Mother	289 (77)
Father	135 (36)
Other Caregiver	23 (6.2)
Roommate	20 (5.7)
My Child/Children	5 (1.3)
Alone	15 (4.1)
My Significant Other	6 (1.5)
Other	45 (12)
Prefer Not to Say	2 (0.50)
**Sibling with SCD in Trial**	
Yes	24 (6.3)
No	351 (94)
**Biological Children**	
Yes	5 (1.3)
Number of Biological Children	1 [1,2]
No	368 (98)
Prefer Not to Say	2 (0.50)
**Primary Insurance Type**	
Public or Government Insurance	227 (61)
Private Insurance	137 (37)
Other	3 (0.70)
None	8 (2.2)
Statistics presented as Number (column %) or Median [min, max]	

### 2.6 Statistical analysis

Descriptive statistics were used to summarize participants' characteristics, recruitment outcomes, and retention rates at 6, 12, and 18 months. Characteristics are presented as counts and percentages for categorical variables and medians with ranges for continuous variables. Retention rates were compared across participant characteristics, recruitment method, site, and intervention arm using chi-square tests. All characteristics are based on the subjects’ baseline values; information on these characteristics was not collected at other time points. For this reason, we report retention by employment status at 6 months only, as this characteristic is likely to change frequently among AYA. All analyses were performed using SAS Enterprise Guide version 8.3, with two-sided tests of hypothesis and a p-value < 0.05 as the criterion for statistical significance.

## 3 Results

### 3.1 Participant characteristics

**Fig. 2:**
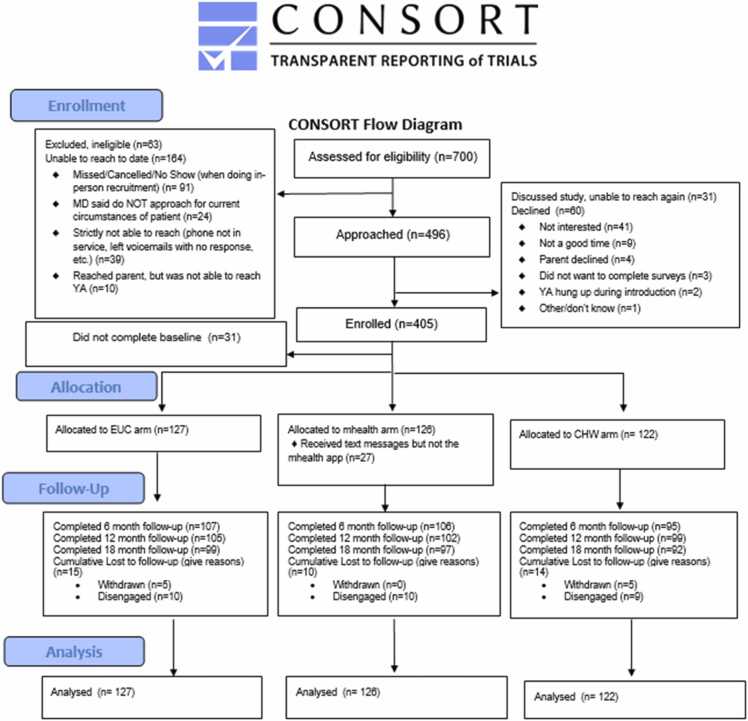
Consort chart.

Of the 700 participants assessed for eligibility for this study, 496 were approached and 405 participants were enrolled. 375 participants were randomized across the five SCD centers: The Children’s Hospital of Philadelphia (CHOP) (n = 143), St. Christopher’s Hospital for Children (St. Chris) (n = 61), Cincinnati Children’s Hospital Medical Center (CCHMC) (n = 37), Cohen Children’s Medical Center (CCMC) (n = 91), and Connecticut Children’s Medical Center (CT Children’s) (n = 43). Participants were randomized 1:1:1 to the intervention arms (CHW: 122, mHealth: 126, EUC: 127) (See Fig. 1, Fig. 2).

Participants’ ages ranged from 17 to 28 years old, with a median age of 18 years. Participants were evenly distributed between identifying as male (49%) and female (50%). A large majority of participants identified as Black or African American (95%), and 8.2% of participants identified as Hispanic/Latino. All participants were characterized as having moderate (43%) or severe (57%) SCD. Full demographic and clinical characteristics can be found in Table 1.

### 3.2 Recruitment outcomes

Patients were primarily identified for trial participation via clinic schedule (83%), with the remaining identified by SCD registry (17%). Subsequently, participants were recruited evenly between in-person contact (51%) and via phone (49%). Of the 700 participants identified for participation, there were 63 ineligible or excluded individuals and 164 that were unable to be reached. This included 91 individuals who either were identified as a missed, cancelled or no show when doing in-person recruitment, 24 individuals where the physician said do not approach for current circumstances of patient, 39 individuals who were strictly not able to reach (phone not in service, left voicemails with no response, etc.) and 10 individuals where we were able to reach a parent, but was not able to reach participant.

Recruitment success in COMETS was supported by close collaboration between RCs and clinical staff. Providers frequently introduced the study or provided a warm hand-off to the RC. Implementing various recruitment strategies (i.e., patient appointments, registry list, in-clinic, and telephone enrollment) created multiple touchpoints for participation (See Table 2).

**Table 2:** Impact of methods on recruitment and retention.

Method	Impact on Recruitment	Impact on Retention
Study introduction by clinic team	High yield	
Same day or next day outreach by RC	High yield	
Multiple touchpoints for recruitment (patient appointments, registry list, in-clinic, and telephone enrollment)	High yield	
Low-risk protocol that did not involve medications or procedures	High yield	
Comprehensive contact information collected at enrollment	Supportive	Supportive
Incentives	Supportive	Supportive
Sustained communication with participant throughout the study		Supportive
Accessibility (ability to complete surveys on smartphones, computers, etc.; ability to pause and return to surveys; in-person study visits not required)	Supportive	Supportive

### 3.3 Retention outcomes

Overall retention rates were 82% at 6 months, 82% at 12 months, and 77% at 18 months. Only 30 participants disengaged from the study after randomization, meaning they completed the initial baseline survey but did not complete any follow-up surveys nor formally withdrew from the study. Patterns of missing data were largely intermittent. Of the 67 participants with missing data at six months, 32 (48%) returned for a subsequent follow-up. Similarly, of the 69 participants missing data at 12 months, 20 (29%) completed the 18-month follow-up, indicating strong longitudinal retention.

Retention varied by site, age, and employment status at select time points (Table 3). Retention at 12 months also differed by age group, with higher retention among participants aged 18 years and younger compared with those 19 years and older. No statistically significant differences in retention were observed across intervention arms at any follow-up point.

**Table 3:** Retention rates stratified by employment, site, age, and recruitment method.

		**6 Months**		**12 Months**		**18 Months**	
**Factor**	**n**	**Number (%)**	**p-value**	**Number (%)**	**p-value**	**Number (%)**	**p-value**
**Site**			0.037		0.0037		< 0.0001
CHOP	143	112 (78)		107 (75)		103 (72)	
St. Chris	61	47 (77)		51 (84)		40 (66)	
CCHMC	37	32 (86)		29 (74)		28 (76)	
CCMC	91	84 (92)		86 (95)		88 (97)	
CT Children's	43	33 (77)		33 (77)		29 (67)	
**Age (at randomization)**			0.82		0.008		0.006
18 and Under	195	161 (83)		169 (87)		161 (83)	
19 and Over	180	147(82)		137 (76)		127 (71)	
**Recruitment Method**			0.69		0.50		0.077
In-person	193	160 (83)		160 (83)		141 (73)	
Via Phone	182	148 (81)		146 (80)		147 (81)	
**Severity Group**			0.52		0.88		0.84
Mild	168	141 (84)		139 (83)		131 (78)	
Severe	197	158 (80)		159 (81)		149 (76)	
**Primary Insurance Type**			0.15		0.41		0.61
Public or Government Insurance	227	179 (79)		184 (81)		172 (76)	
Private Insurance	137	120 (88)		114 (83)		109 (80)	
Other	3	3 (100)		3 (100)		2 (67)	
None	8	6 (75)		5 (63)		5 (63)	
**Intervention Arm**			0.33		0.93		0.89
Community Health Worker	122	95 (78)		99 (81)		92 (75)	
Mobile Health Application	126	106 (84)		102 (81)		97 (77)	
Enhanced Usual Care	127	107 (84)		105 (83)		99 (78)	
**Employment**			0.040				
Employed	57	43 (75)					
Student	133	117 (88)					
Student and Employed	76	64 (84)					
Other	100	79 (79)					

Statistics presented as N (column %). P-values are Pearson’s chi-square test

## 4 Discussion

The COMETS study achieved strong recruitment and retention outcomes, demonstrating the effectiveness of its various strategies to engage AYA with SCD. Despite the challenge of the COVID-19 pandemic, this 18-month study successfully enrolled 405 participants exceeding our recruitment goal, of whom 375 were randomized. Attrition was notably low, with only 30 participants disengaging after completing the baseline survey.

There remains limited research examining recruitment and retention approaches tailored to AYA with SCD. Prior studies have shown that multiple methods of recruitment support successfully enrolling patients into research studies.^13^, ^14^ This study adds to the growing body of work by identifying adaptable, participant-centered methods for additional strategies recruiting and retaining AYA with SCD in a longitudinal study.

A main driver of recruitment success in the COMETS study was the identification and approach of patients via in-person clinic visits. This finding is consistent with literature on a behavioral health trial for AYA with overweight and obesity, in which clinic recruitment was found to be a highly successful strategy in acquiring participants, especially in underrepresented groups.^15^ The impact of clinic visits in driving diverse participant recruitment highlights the importance of utilizing existing frameworks and clinician-patient relationships to identify AYA for study participation.

A central strength of COMETS was its patient-centered design. The study consisted of a stakeholder advisory committee (SAC), which included AYA patients as key stakeholders. The AYA patient stakeholders were influential in the study’s final compensation values. The study showed significant retention by employment status (p = 0.040). Studies have shown that even if there is interest in participating in research, money can be a barrier or a facilitator in one’s decision to participate.^16^, ^17^ The SAC met regularly throughout the study to review progress, identify challenges, and celebrate success through ongoing meetings and newsletters.

Additionally, weekly meetings between Research Coordinators across the five participating sites and study program managers allowed for a continuous feedback loop for monitoring recruitment and retention. These meetings facilitated shared learning, allowed for site-level problem solving, and enabled the research team to refine strategies in real time. These meetings may have supported site-level retention by facilitating shared learning, problem solving, and real-time strategy refinement. Consistent with prior evidence, the COMETS study’s success reflects the combined use of proactive recruitment approaches (e.g. direct, in-person engagement) and reactive methods (e.g. videos, flyers).^13^ Strong retention rates were likely bolstered by a clearly defined survey workflow tailored to the extended 18-month participation period, strategic use of incentives, and the study team’s swift adaptation to virtual engagement during the COVID-19 pandemic.

### 4.1 Limitations

The COMETS study launched in January 2019 with the expectation that the study’s mobile health app would be operational shortly thereafter. However, technical delays required a study pause until the app was completed. The pause lasted from May 2019 and was extended to July 2020 due to the COVID-19 pandemic. Participants randomized to the mobile health arm were therefore analyzed using an intention-to-treat approach. The onset of the COVID-19 pandemic presented an additional and unprecedented challenge. National stay-at-home orders reduced healthcare utilization,^14^ significantly limiting opportunities for in-person recruitment. In response, the COMETS team implemented several adapted measures, most notably, electronic consent and new workflows for virtual enrollment and participant communication. These adaptations were consistent with other pandemic-era research practices, including the use of HIPAA-compliant e-consent and secure SMS reminders.^10^ However, COVID-19 recruitment strategies that have since been described post-pandemic, such as targeted social media campaigns, might have further expanded recruitment reach.^14^ Nevertheless, the COMETS team demonstrated considerable flexibility and innovation in maintaining enrollment and engagement during a period of profound disruption.^18^

## 5 Conclusion

The COMETS study demonstrates that participant-centered, multi-modal strategies, grounded in strong clinical partnerships, can yield high recruitment and retention rates among AYA with SCD, even amid substantial external challenges. These findings underscore the value of flexible, relationship-based approaches in sustaining engagement in longitudinal research for those living with a chronic disease.

## CRediT authorship contribution statement

**Abena Appiah-Kubi:** Writing – review & editing, Project administration. **Robin Arens:** Writing – review & editing. **Amy Shova:** Writing – review & editing. **Steinway Caren:** Writing – review & editing, Supervision, Project administration, Methodology, Conceptualization. **Mark Ferreira:** Writing – review & editing. **Sophia Jan:** Writing – review & editing, Supervision, Project administration, Conceptualization. **Amanda Pfeiffer:** Writing – review & editing. **Kim Smith-Whitley:** Writing – review & editing, Supervision, Project administration, Conceptualization. **Laura Bennett:** Writing – review & editing, Formal analysis. **Omar Niss:** Writing – review & editing, Supervision, Conceptualization. **Olivia Teng:** Writing – review & editing, Writing – original draft, Project administration. **Donna Boruchov:** Writing – review & editing, Supervision, Project administration. **Bianca Ferreira:** Writing – review & editing, Writing – original draft. **Nataly Apollonsky:** Writing – review & editing, Project administration. **Abigail Seide:** Writing – review & editing, Writing – original draft. **Evelyn Stevens:** Writing – review & editing, Writing – original draft. **Banu Aygun:** Writing – review & editing, Project administration. **Lane Carbaugh:** Writing – review & editing, Writing – original draft. **Williford Desireé:** Writing – review & editing. **Tanisha Belton:** Writing – review & editing, Writing – original draft, Supervision, Project administration, Conceptualization.

## Ethical statement

We attest that this work has been carried out in accordance with the Code of Ethics of the World Medical Association (Declaration of Helsinki) and is aligned with the Recommendations for the Conduct, Reporting, Editing, and Publication of Scholarly Work in Medical Journals.

## Funding

This work was funded through a Patient-Centered Outcomes Research Institute (PCORI) Award (MCSC-1609–36631). The statements in this manuscript are solely the responsibility of the authors and do not necessarily represent the views of the Patient-Centered Outcomes Research Institute (PCORI), its Board of Governors or Methodology Committee.

## Declaration of Competing Interest

Kim Smith-Whitley initiated this project as a full-time Perelman School of Medicine University of Pennsylvania employee and Director of the Comprehensive Sickle Cell Center at Children’s Hospital of Philadelphia. She transitioned to Professor Emeritus and Physician Affiliate at CHOP in May 2021 with Global Blood Therapeutics and in September 2022 with employer Pfizer. Biree Andemariam received consultant fees from Accordant, Afimmune, Agios, Beam Therapeutics, bluebird bio, Chiesi, Editas, Genentech, GlaxoSmithKline, Hema Biologics, Hemanext, Merck, Novartis, Novo Nordisk, Octapharma, Pfizer, Roche, Sanius Health, Sanofi Genzyme, Vertex. Research support from Afimmune, Agios, American Society of Hematology, American Thrombosis Hemostasis Network, Centers for Disease Control, Connecticut Department of Public Health, Hemanext, Health Resources and Services Administration, Novo Nordisk, Patient-Centered Outcomes Research Institute, Pfizer and data safety monitoring support from Editas and Fulcrum Therapeutics. Omar Niss reported receiving advisory board fees from Pfizer Inc outside the submitted work. Banu Aygun received grant support from Bluebird Bio Inc and Pfizer Inc and personal fees from Global Blood Therapeutics Inc, Pfizer Inc, and Agios Pharmaceuticals Inc outside the submitted work. Abena Appiah-Kubi received grant support from Novo Nordisk A/S and the Doris Duke Foundation, advisory board fees from Pfizer Inc and Chiesi Farmaceutici SpA, and consulting fees from the office of Federal Public Defender outside the submitted work. All other authors declare that they have no conflict of interest, including affiliations with or involvement in any organization or entity with any financial interest or nonfinancial interest in the subject matter or materials discussed in this manuscript.

## Data Availability

Data will be made available on request.
